# Reframing the significance of menstruation: evolutionary insights from an organismal-relational perspective

**DOI:** 10.1007/s40656-025-00710-5

**Published:** 2025-12-16

**Authors:** Arantza Etxeberria, Ainhoa Rodriguez-Muguruza

**Affiliations:** IAS Research Group for Life, Mind and Society, Department of Philosophy, University of the Basque Country, EHU, Donostia/San Sebastian, Gipuzkoa, Spain

**Keywords:** Evolutionary medicine, Menstrual cycle, Adaptation, Stigma, Evo-devo, Organismal approach, Modern Synthesis, Extended Synthesis

## Abstract

Cultural stigma and medical pathologization have long shaped scientific and social perceptions of menstruation, limiting both research and clinical attention. This paper outlines three major sources of negative perceptions and examines their influence on scientific discourse and cultural attitudes. To counter these biases and misconceptions, evolutionary accounts of menstruation are explored, which emphasize its crucial role in human physiology and reproduction. Two evolutionary approaches to adaptation are compared: one adopts a functionalist stance that assigns specific functions to traits. While this perspective offers a naturalized and positive understanding of menstruation, it remains insufficient to capture the phenomenon’s full complexity. In response, the paper draws on a second approach, organismal and relational, which emphasizes whole-organism adaptation within developmental and ecological contexts. This contrast is also reflected in evolutionary medicine, where organismal approaches support integrative views of disease patterns. Revisiting late 20th-century debates on whether menstruation is adaptive or a byproduct, the paper presents key elements of the alternative organismal-relational perspective. This framework makes it possible to distinguish three broad categories of menstrual pathologies and supports the claim that organismal evolutionary perspectives offer a richer understanding of menstrual health.

**Supplementary Information:**

The online version contains supplementary material available at 10.1007/s40656-025-00710-5.

## Introduction

Menstruation is a fundamental process that engages deeply with core questions of human experience, revealing the close relationships between bodily processes and their social and environmental relational contexts. Despite its relevance, it remains underexplored in the philosophy of science, particularly in key areas such as the philosophy of biology and medicine. Moreover, menstruation is often negatively perceived due to persistent stigma, widespread misconceptions, and its longstanding medical pathologization (Olson et al., 2022). This lack of attention, coupled with prevalent misunderstandings, calls for a reassessment of menstruation from a broader and more integrative perspective. To this end, we argue that understanding the evolutionary significance of menstruation can help address these issues by providing both scientific and philosophical insights. A key question we address is which evolutionary framework best accounts for what menstruation is, how it evolved, and the role it plays in the health and well-being of menstruating individuals. In this paper, terms like *“menstruating bodies,” “menstruating organisms,”* *“menstruating individuals,”* or *“menstruators,”* are used to emphasize the physiological and ecological dimensions of menstruation without conflating them with fixed sex categories. While menstruation is physiologically connected to reproductive cycles in certain mammalian lineages and in individuals with uterine cycles, this terminology helps maintain focus on the phenomenon itself rather than on essentialized group identities.^1^

Even among mammals, menstruation is a rare phenomenon, observed in only a few phylogenetically diverse groups, including the elephant shrew, certain rodents (such as the spiny mouse), a few species of bats, and some primates, including humans. Interestingly, menstruation is believed to have evolved independently on at least four occasions and is currently observed in only about 1.6% of extant eutherian mammal species (Günter Wagner, in Critchley et al., 2020, p. 629). As a distinctive phase within a complex physiological system, menstruation invites us to reconsider how reproductive cycles are organized and understood. While most mammals have distinct phases of sexual receptivity and fertility, most of them do not exhibit *true* menstrual cycles, as vaginal bleeding in the absence of endometrial shedding does not constitute menstruation per se. The occurrence of menstrual bleeding is evolutionarily contingent on other developmental traits, such as spontaneous ovulation and uterine decidualization,^2^ features often mentioned in definitions of menstruation,^3^ and which will be examined later in the paper.

A recurring question, raised in philosophy of science and broader interdisciplinary discussions, is why menstruation exists and whether it is necessary (Jarrell, 2018; Renfree, 2012). To contribute a philosophically grounded account of its significance, our strategy is to draw on evolutionary explanations of menstruation to challenge misconceptions, particularly given its rarity and the myths that surround it (Critchley et al., 2020). While the topic has received limited philosophical analysis, it began to attract broader interdisciplinary attention toward the end of the twentieth century.

In particular, a central debate emerged in the evolutionary literature, which was echoed and discussed in the philosophy of science. It concerns whether menstruation serves a biological function or is merely a byproduct arising when other traits have been selected, with implications for how menstrual health is understood and treated (Clough, 2002; Howes, 2010). While early adaptationist theories sought specific functions (Haig, 1993; Profet, 1993), critical approaches emphasized menstruation as part of a broader system of reproductive and physiological processes (e.g. Finn, 1994; Strassman, 1996). More recently, researchers have begun to view the entire menstrual cycle as a systemic evolutionary phenomenon shaped by developmental and ecological factors, one that resists simple straightforward explanations focused solely on cyclical bleeding (Emera, 2023, Rodríguez-Muguruza, 2023; Martínez-Quintero & Rodríguez-Muguruza, 2025). This shift in perspective invites renewed philosophical reflection and has consequences for how menstrual health is conceptualized within evolutionary medicine.

In this paper, we claim that evolutionary accounts of menstruation can deepen our understanding of its biological significance and its implications for menstrual health and disease, provided that appropriate theoretical approaches are adopted. We begin by reviewing some of the historical and cultural sources underlying negative views of menstruation (Sect. 2). Then, we introduce two approaches to explaining the evolution of biological traits: one grounded in a functional-adaptive framework, and another emerging from more recent organismal, developmental, and ecological thinking (Sect. 3). These frameworks set the stage for the following sections. We look first at the late 20th-century debate around whether menstruation has a specific evolutionary function or whether it might be better understood as a byproduct of other processes (Sect. 4). We then turn to new research that frames menstruation within a developmental, sytemic and relational understanding of organisms (Sect. 5). Building on this, we examine how different evolutionary features of menstruation can shed light on why certain menstrual pathologies occur (Sect. 6). We conclude by bringing together the key insights developed throughout the paper to consider what they reveal about the evolutionary reasons why some individuals menstruate, and why this matters for understanding their health and care.

## Negative views of menstruation and pathologization

Menstruation has long been burdened with negative connotations (Bobel et al., 2020). This section examines three key misconceptions about menstruation including stigmatizing attitudes, certain interpretations within evolutionary theory, and entrenched medical assumptions. Together, these perspectives have hindered a more accurate and integrative understanding of menstruation accross science, medicine, and philosophy.

The first one is rooted in social stigma, which manifests through taboos, stereotypes, and discrimination: menstruation has often been framed in harsh, disparaging terms, with some even comparing it to toxic waste (Johnston-Robledo & Chrisler, 2020). Such attitudes fuel perceptions of menstruating individuals as unwell, lacking control, or mentally unstable (Chrisler, 2008; Chrisler & Caplan, 2002; Johnston-Robledo & Chrisler, 2020), reinforcing not only the pathological framing of menstruation itself but also the stereotyping and medicalization of the individuals who experience this developmental stage. While contemporary medicine generally recognizes menstruation as a constitutive physiological process within some life trajectories, it has historically been framed as a site of disadvantage, associated with inferiority and subjected to belittlement.

In this context, “pathologization” does not necessarily mean that menstruation is formally classified as a disease. Rather, its symptoms and effects have often been interpreted through a framework of deficiency or devaluation. Within this view, menstruation is regarded primarily for its instrumental role in signaling pregnancy, rather than as a meaningful physiological process in its own right. This framing is problematic for at least two reasons. First, it relies on a normative model of health that centers male bodies, historically treated as neutral or universal, as the standard,^4^ thereby rendering menstruation and other cyclical processes as deviations from that norm. Second, this view oversimplifies its biological complexity neglecting the wide variety and diversity of menstrual experiences, and reinforces cultural stigma (Bobel et al., 2020; Guilló-Arakistain, 2020). It encourages an interpretation of menstruation as a problem to be managed or corrected, rather than as a meaningful and functional physiological process.

Philosophers like Simone de Beauvoir (2011) and Iris Marion Young (2005) have explored how social norms shape the perception of menstruation. Beauvoir famously described menstruation as repugnant and degrading, a characterization critics have described as “somatophobic” (Groenhout, 2017, p. 73). In contrast, Young’s phenomenological “menstrual meditations” highlight the secrecy, shame, and exclusion menstruators experience, particularly in institutional settings like schools and workplaces. While Young sought to cast menstruation more positively, she ultimately concluded that its social and embodied experiences remained largely constrained. These accounts underscore how stigma becomes internalized, shaping menstruators’ own perceptions and, at times, leading to unnecessary medical interventions (Chrisler, 2011; Ussher, 2006; Ussher & Perz, 2013). Their philosophical premises point to the depth of menstrual stigma, even in feminist thought, and raise the question of whether an evolutionary perspective might offer a more neutral, biologically grounded way of understanding menstruation that avoids culturally imposed normative assumptions.

A second example reveals that menstruation has been characterized as problematic not only by philosophers, but also by scientists; certain strands of evolutionary biology appear to be tainted by underlying stigma. One example is the outdated “handicap” principle, suggesting menstruation is a costly signal of health for sexual selection. According to some evolutionists, certain physiological processes function as signals among organisms; those signals are considered to be “handicaps*”* because they are costly to produce and, as such, they are disadvantageous for adaptive purposes. Menstruation, in this context, is considered a “handicap”, a signal incurring fitness costs yet potentially conferring advantages in sexual selection (Zahavi & Zahavi, 1999).

A related concept is “mismatch”, which proposes that the current patterns of menstruation are misaligned with the evolutionary conditions of our ancestors. It highlights how the frequency of menstruation today is much higher than it was in ancestral times. Although this framework has been used to explain a variety of modern health issues (Bourrat & Griffiths, 2024; Morris, 2020), its application to menstruation has sometimes reinforced the idea that the phenomenon is inherently dysfunctional. Here we briefly signal this limitation, not to reject the mismatch perspective outright, but to indicate that its explanatory scope depends on how the relation between organism and environment is conceived, a point we return to in Sect. 6, where we offer an organismal reinterpretation of mismatch based on more systemic, developmental, and relational insights. While these evolutionary accounts have been influential, they risk reinforcing the idea that menstruation is currently dysfunctional, without providing evidential explanations for why this phenomenon should be regarded as inherently problematic.

A third instance concerns medical interpretations of menstruation, particularly the tendency to regard inflammation as inherently pathological. In modern biomedicine, inflammation is commonly understood as a sign of infection or chronic disease. However, in the context of menstruation, inflammatory processes fulfil essential physiological functions, including tissue repair and preparation of the endometrium for potential pregnancy. In fact, processes like menstruation, embryo implantation, and childbirth naturally involve inflammatory responses. This connection has been recognized since at least the late nineteenth century, when decidual cells, part of the uterine lining, were described as “phagocytes” for their active role in these processes (Finn, 1986). This association, alongside the pain and discomfort often experienced during menstruation, has contributed to the paradoxical perception of it as a potentially disordered condition. Yet this is not, in principle, the case: recent research suggests that menstrual inflammation serves a physiological and functional role, rather than representing a pathological response (Chavan et al., 2017, p. 24). This view aligns with the growing recognition that inflammation is not solely a detrimental process but also an essential part of the body’s response to various processes (Medzhitov, 2008). Beyond their ultimate role in reproduction, these inflammatory processes also fulfil important physiological functions related to the cyclical preparation and renewal of reproductive tissues, even in the absence of pregnancy.

A particular aspect of menstruation that has attracted considerable attention is menstrual pain, or dysmenorrhea. This phenomenon, experienced by the majority of menstruating individuals, raises important questions regarding its origins, inevitability, and evolutionary significance. Menstrual pain has been linked to the relatively recent evolutionary emergence of menstruation, where the evolutionary benefits of menstruation (e.g., reproductive strategy) may not fully offset the physiological costs (e.g., pain from uterine contractions) (Serrahima & Martínez, 2023). This pain, connected to increased uterine contractility, may be functional for effectively clearing the uterine cavity. Parallels between menstrual and birthing contractions suggest that both are triggered by similar mechanisms related to parturition (Pavliçev & Norwitz, 2018). Thus, strong menstrual contractions are sometimes seen as an indication of heightened fertility (Leyendecker & Wildt, 2019). This argument reinforces the perception of menstrual pain as inevitable, further solidifying its association with suffering and negativity.

In sum, philosophical, societal, evolutionary, and medical perspectives have often treated menstruation as a biological challenge or irregularity. However, recent work in evolutionary and developmental biology, immunology, and feminist philosophy offers alternative frameworks that emphasize its functional and evolved nature. By reinterpreting menstruation as an integral part of human physiology, rather than a likely source of dysfunction, we can foster a more nuanced understanding and support more responsive and inclusive healthcare approaches.

## Evolutionary frameworks: functionalist vs. organismal approaches

The question “What is menstruation for?” offers an insightful framework for examining its biological and medical relevance through its evolutionary history. This issue sparked considerable debate in evolutionary biology and medicine in the 1990s. Early scholars addressing the evolutionary causes of menstruation were often motivated by the need to vindicate and dignify it as a meaningful biological phenomenon. Rather than viewing menstruation as useless or accidental, they argued that it has important evolutionary foundations that warrant serious scientific attention (Rogers-LaVanne & Clancy, 2021). However, these efforts soon became entangled in broader theoretical debates within evolutionary biology, which also influenced evolutionary medicine.

For our purposes, we can distinguish two main frameworks that offer contrasting ways of understanding the evolution of traits, including menstruation. The first is a functionalist perspective, which tends to explain characters primarily in terms of adaptive value and natural selection. The second can be described as organismal, aligning more closely with structuralist and ecological approaches that emphasize the role of form, developmental constraints, and the organism’s agency in shaping evolutionary outcomes.

The functionalist approach falls, broadly speaking, within what is known as the Modern Synthesis (MS), a framework for understanding biological phenomena through an evolutionary perspective that emerged in the first half of the twentieth century through the integration of Darwinian natural selection, population-level thinking, and Mendelian inheritance. The MS has been the dominant conceptual framework for evolutionary biology since the 1940s, holding that natural selection is the main driver of adaptation and aiming to explain traits through their functional alignment with the environment. Thus, it emphasizes adaptation by focusing on how traits enhance an organism’s fitness. This framework typically prioritizes explaining biological traits in terms of their function or contribution to survival and reproductive success.

The organismal approach, by contrast, arises from a series of challenges to the MS, emerging mainly from developmental biology and ecological perspectives. These approaches emphasize structuralism and the idea that organisms are active agents in evolution (Brown, 2022; Etxeberria, 2023; Cortés García & Etxeberria, 2023; Fábregas-Tejeda et al., 2024; Nuño de la Rosa, 2023). In particular, they question the preeminence of natural selection in explaining adaptation, arguing instead that developmental processes also play a crucial role in shaping evolutionary outcomes, influencing both the direction and pace of evolution (Gould & Lewontin, 1979; Novick, 2023).

Unlike functionalism, structuralism focuses on organic form; it emphasizes the role of internal mechanisms, such as developmental constraints and biases, in shaping phenotypes. Thus, traits do not arise from direct adaptive pressures; rather, they emerge through developmental processes that are independent of, and precede, natural selection (Brown, 2022). The form of an organism is strongly shaped by developmental processes, suggesting that not all traits necessarily have an adaptive function primarily driven by natural selection. Instead, these traits may arise from developmental constraints and biases that influence their expression, independent of selective pressures. Moreover, organisms are active agents that construct their own environments and shape their selective pressures (niche construction theory), and developmental trajectories (Evolutionary Developmental Biology or Evo-Devo). The organismal approach therefore emphasizes causal reciprocity between organisms and their environments and highlights the importance of relational accounts (Baedke et al., 2021; Barandiaran & Etxeberria, 2026; Etxeberria, 2020). Together, these insights have contributed to the development of the more encompassing evolutionary framework, known as the Extended Evolutionary Synthesis (Laland et al., 2015; Müller, 2017), which criticises the MS on key issues such as the origins of variation, the organism-centered framework, and the role of plasticity in evolutionary processes (Nuño de la Rosa & Müller, 2021). The functionalist and organismal approaches offer different explanatory strategies, and together may contribute to a richer understanding. While these views were in stark opposition in the late twentieth century, today evolutionary fields are shifting toward more integrative perspectives (Brown, 2022; Novick, 2023).

This dual perspective in evolutionary biology is also mirrored in evolutionary medicine. In this field, it has become increasingly evident that approaches closely aligned with the MS often provide limited explanations, prompting the need for deeper insights from developmental biology and ecological relationships. The integration of evolutionary biology into medicine was proposed by scholars such as George C. Williams and Randolph M. Nesse, who called for the incorporation of evolutionary concepts into medical theory and practice (William & Nesse, 1991). Initially known as *Darwinian medicine* (DM), this approach adopted a functional, adaptationist framework, and argued that since human bodies have been *designed* by natural selection to adapt to their environments, it is problematic for medicine to overlook evolutionary concepts. For Mayr, proximate causation characterized fields like medicine, which traditionally did not appeal to evolutionary, or ultimate, explanations (Mayr, 1961). Proponents of DM argue that explanations of pathological states require integrating ultimate causes into medical thinking and to incorporate evolutionary concepts previously absent from medical curricula.

The adaptationism of DM faced substantial criticism for emphasizing ultimate explanations that, while theoretically valuable, may lack immediate clinical relevance (Cournoyea, 2013, 2016; Méthot, 2011; Vallés, 2012). Pierre-Olivier Méthot, for instance, distinguishes DM from a more general conception of Evolutionary Medicine (EM), which he argues is a tradition that predates DM by several decades and which is characterized less by a focus on adaptation and more by a diversity of research agendas (Méthot, 2011 ). Méthot further contends that, although DM constitutes a research tradition unified by certain theoretical and methodological principles, it faces several limitations. These include its focus on evolutionary history, a narrow conception of adaptation, and a lack of practical guidance for clinical application. In contrast, he suggests that a broader version of EM real-time evolutionary processes, offers a complementary and valuable perspective. In this broader sense, evolutionary theory serves as an additional research tool for medical researchers, clinicians, health professionals, and policymakers seeking practical solutions to urgent medical problems (Natterson-Horowitz, 2023).

Drawing on this work, Nina Kranke argues that EM, unlike DM, does not have strong internal cohesion in terms of epistemology and methodology (Kranke, 2023). As the association is loose, there are no commonly agreed-upon goals or approaches for interdisciplinary or explanatory integration within the field. This feature of EM is valuable for its ability to generate genuinely integrated explanations and its clinical relevance. While DM is characterized by the use of adaptationism as a heuristic principle in medicine, according to Kranke, EM is more than a heuristic, it involves the direct application of methods, practices, and concepts from evolutionary biology to address medical problems. In this context, Evolutionary Medicine (EM) may appear less internally cohesive than the Modern Synthesis–based approaches, since it draws from multiple biological and clinical traditions rather than a single theoretical core. Yet this very pluralism allows it to generate more integrated explanations, linking molecular, physiological, ecological, and social dimensions of health. Interdisciplinarity, in this sense, is not a sign of theoretical weakness but a productive condition for developing organism-centered frameworks of explanation.

As we will see, both functionalist and organismal approaches have been applied to explain the evolution of menstruation, though they differ significantly in their explanatory scope and medical implications. Early DM-like adaptationist theories primarily sought to explain why menstruation evolved, yet these approaches often failed to account for the underlying physiology and complex biological interactions involved, providing limited insight. In contrast, EM-like explanations examining how new intra- and inter-organismal relations relevant to menstruation emerge, particularly within the evolution of the maternal–fetal interface (including endometrial development, specialized immune cells, and hormonal transformations), provide a more substantial basis for understanding why menstruation occurs in some species (Cortés-García et al., 2024). By comparison, functionalist approaches grounded in adaptationism provide limited explanatory power compared to the organismal perspectives offered by EM (Cortés-García & Etxeberria, 2023).

The distinction between functionalist and organismal perspectives has significant implications for understanding the evolutionary significance of menstruation. The former tends to emphasize fitness advantages for survival or reproduction, whereas the latter considers the role of menstruation within broader physiological and ecological systems. The question of ‘why’ menstruation evolved is embedded in the ‘how’ it manifests within the integrated organism. This may contribute to better assessing its medical and social implications. Factors such as pain and bleeding may be better understood as outcomes of integrated systems shaped by evolutionary dynamics, offering deeper insight than explanations based solely on proximate causes.

In the following sections, we review research on the evolution of menstruation, first by examining the history of adaptive vs. byproduct debates from the 1990s, and then organismal perspectives that align with the EM framework. We argue that organismal approaches offer richer medical and conceptual insights than traditional functionalist adaptationist accounts.

## Functional and byproduct hypotheses on menstruation

In the late twentieth century, a major debate emerged concerning the evolution of menstruation, focusing primarily on its functionality. The key question was whether menstruation has its own evolutionary origin and function, that is, whether it evolved to fulfil a specific adaptive role. As previously noted, this debate was particularly compelling because its proponents sought to demonstrate that menstruation was not a biologically purposeless trait, or a “defect” (Profet, 1993, p. 336), but rather one with an evolutionary function. In doing so, their argument aligned with the dominant perspective in evolutionary biology at the time, which maintained that traits are considered functional if they possess a clearly identifiable role tied to their original adaptive significance. From this standpoint, only those characters with historical adaptive roles were regarded as functional.

One of the possible triggers for the discussion may have been when Colin A. Finn, a veterinarian researcher, posed the question “Why do women and some other primates menstruate?” (Finn, 1987). He answered that menstruation is an inevitable consequence of a complex reproductive system and does not serve a distinct function on its own. Finn explores historical and modern understandings of menstruation, tracing ideas from ancient beliefs about its cleansing function to modern scientific theories that link it to the ovarian cycle and the preparation of the uterus for implantation. He also examines differences in embryonic implantation across species and suggests that menstruation is a consequence of the advanced preparation of the endometrium in primates and other menstruating groups; this preparation is essential for implantation but results in the breakdown of the tissue if pregnancy does not occur. Therefore, menstruation would be the inevitable result of those endometrial modifications, rather than a process with an independent primary physiological function. Although the immediate (proximate) cause of bleeding is a drop in progesterone levels, according to Finn, the true evolutionary cause lies not in the ovary but in the aforementioned uterine processes. Bleeding, he maintains, is a non-adaptive byproduct of uterine evolution (Finn, 1994; Martin, 2007).

In the meantime, several hypotheses were proposed to explain menstruation as an adaptive function, suggesting that it enhances survival, reproduction, or fitness. One of the most controversial ones was put forward by Margie Profet, who proposed that menstruation evolved as a mechanism to eliminate pathogens and bacteria introduced to the uterus by spermatozoa (Profet, 1993). In fact, earlier interpretations that portrayed menstruation as an inefficient bodily process, namely, one that discards rather than recycles the nutrient-rich uterine lining, were critically reassessed in Profet’s work. She claims that uterine shedding may occur even in species without visible external menstruation, with reabsorption rather than expulsion as a possible outcome. In this view, both internal (covert) and external (overt) menstruation are thought to serve the same primary function: defending against pathogens introduced by sperm. Accordingly, menstruation serves the specific adaptive function in mammals of protecting the uterus and fallopian tubes against infections that may be introduced during insemination, menstrual bleeding is not an inefficient byproduct of the reproductive cycle, but rather an active mechanism shaped by natural selection to protect female reproductive health. Profet’s hypothesis explicitly treated menstruation as a discrete evolutionary adaptation, isolating it evolutionarily. Physiologically, however, her account did not treat menstruation as independent of other bodily systems as it invoked immune, vascular, and hormonal processes working together to achieve the presumed defensive role.

Overall, Profet disputes the byproduct views of Finn and others, and supports functionality by drawing on the adaptationist framework developed by Williams and Nesse in the context of DM or Darwinian medicine. She holds that when adaptive explanations are overlooked, biological processes such as menstruation risk being misunderstood as flaws or defects (p. 336). That is why she seeks explanations that demonstrate menstruation is not merely a loss, thus building on evolution as adaptation (Williams, 1966) and the principles of early Darwinian medicine (Williams & Nesse, 1991), Profet suggested that menstruation’s nutritional and reproductive costs serve as an anti-pathogenic function and menstruation is neither costly nor useless (Clough, 2002).

While Profet’s hypothesis gained initial attention, it also faced criticism. Critics argued that her approach oversimplified the complexity of the reproductive system and isolated menstruation from broader evolutionary processes. Her controversial hypothesis drew significant scientific attention, as she urged researchers to consider menstruation’s role in other organic systems, raising awareness over the risk of neglecting the view of the organism as a holistic physiological system (Rako, 2003). By questioning the neglect of functional causes, Profet aimed to reposition menstruation within evolutionary biology, emphasizing its significance for human physiology (Clough, 2002, see also Placek, 2021 on Margie Profet´s life and scientific contributions). Following Profet’s hypothesis, other adaptationist theories were proposed, suggesting that menstruation may be related to conflicts between the gestating parent and embryo (Haig, 1993) or act as a mechanism for eliminating defective embryos (Clarke, 1994).

Critics of adaptationist explanations expanded their objections in subsequent work. After the initial paper, Finn in particular reaffirmed his view that menstruation lacks an adaptive explanation, arguing instead that it is an accidental consequence or byproduct of other processes (Finn, 1994, 1996, 1998). For instance, Finn mocks the idea that menstruation evolved to combat sperm-borne pathogens, noting instead that it occurs as a “response to the absence of spermatozoa” (Finn, 1994, p. 1202). He stressed the importance of studying non-menstruating species, many of which are closely related to those that menstruate, to gain a clearer understanding of how menstruation evolved. Finn maintains that menstruation emerged as a byproduct of spontaneous decidualization, an evolutionary novelty: unlike in most species, where uterine decidualization is triggered by embryo implantation, in menstruating species the endometrium is cyclically lined with decidual cells regardless of the presence of a blastocyst. He also uses the argument that menstruation with repeated ovulation is not common in many non-Western societies, making it difficult to consider that it has the function described by Profet.

Further elaborations on this aspect of the debate also come from Beverly Strassmann, an evolutionary anthropologist who proposes that menstruation results from endometrial regression (Strassman, 1996). According to Strassmann, this regression process, rather than being excessively costly, actually conserves energy compared to the sustained maintenance required to keep the endometrium in a differentiated state for implantation. She viewed menstrual bleeding as a non-adaptive consequence of endometrial regression. While the cyclical shedding of the endometrium prepares the uterus for potential implantation, the bleeding itself is considered an unintended side effect: menstrual bleeding is not functional, but rather a byproduct of the process of endometrial renewal. This renewal is part of a broader biological process, with the goal not necessarily being the expulsion of uterine tissue, but rather the preparation of the uterus for potential implantation. However, Strassman acknowledges that endometrial renewal, along with its accompanying hormonal activity, plays a key role as it facilitates uterine receptivity and regulates the reproductive cycle. Though she doesn’t assign a direct purpose to the bleeding itself, the cycle of endometrial renewal and its supporting dynamics are essential for understanding how menstruating bodies prepare for possible conception.

This debate illustrates how, during the 1990s, questions about whether menstruation has an evolutionary function were framed within a broader theoretical context in which “function” was largely understood in terms of natural selection. Adaptive hypotheses argued that menstruation evolved to serve a specific function that allegedly conferred an advantage in ancestral environments. Several proposals have been put forward in this regard, including the removal of pathogens, the signalling of fertility to potential mates, and the expulsion of defective embryos (Profet, 1993; Clarke, 1994). In contrast, other scholars were wary of adaptive explanations and instead focused on proximate causes to explain their evolutionary relevance (Finn, 1996 ; Strassmann, 1996). These scholars argue that understanding the physiological complexities of menstruation is essential to account for its functionality, emphasizing the causal roles of parts within a broader system rather than attributing a direct adaptation to each individual feature. These contrasting views offer different perspectives on menstruation’s evolutionary significance. While adaptive positions often defend the necessity of menstruation, and byproduct positions regard it as incidental (Rogers-LaVanne & Clancy, 2021), neither perspective deems it “useless.” Therefore, we emphasize that the disagreement lies in the theoretical and philosophical approaches to how evolutionary biology and medicine can explain functions. The organismal perspective developed in the next section helps to reframe how functions are understood in relation to the living body as a whole, rather than as isolated traits shaped solely by adaptation.

Another source of disagreement and misunderstanding in this debate has been the discussion surrounding the potential health benefits or risks of suppressing menstruation. While neither side of the evolutionary debate provides definitive support for or against its suppression across the entire population of menstruators, the issue has prompted broader questions. Some suggest that eliminating menstruation could benefit the health of menstruators, mainly based on mismatch arguments that we elaborate later in Sect. 6 (Howes, 2010), particularly noting that modern individuals experience far more menstrual cycles than ancestral populations due to fewer pregnancies and longer lifespans. By contrast, scholars like Clarke (1994), Martin (2007), Profet (1993), and Rako (2003) argue that menstruation plays a crucial role in maintaining physiological health, with its persistence in human physiology signaling its importance. As such, they caution against attempts to suppress it (Coutinho & Segal, 1999; Hitchcock, 2008). Nevertheless, neither the adaptive nor the byproduct explanations for the evolution of menstruation offer conclusive evidence to support or oppose suppression (Valles, 2012), indicating that other considerations are needed.

## Evolution of menstruation from an organismal perspective

Since the debates of the 1990s, discussions about the evolutionary significance of menstruation have shifted toward what we describe as an *organismal turn*. Research from recent decades can be reframed within this renewed approach, which emphasizes the integrated organization of physiological and developmental processes, while also acknowledging their ecological and social relational dimensions. Rather than reducing menstruation to isolated functions, this perspective situates evolutionary adaptation within the broader context of the whole organism, embedded in its relational environment. Finn connected the evolution of menstruation to the increasing invasiveness of placentation, suggesting that the coevolution of the embryo and the gestator’s reproductive tract necessarily involved adaptation of the inflammatory response (Martin, 2007). This insight influenced subsequent scholars, including Strassman (1996), Emera et al. (2012), and Critchley et al. (2020), and contributed to a broader shift away from strictly functionalist interpretations of menstruation.

More recent studies emphasize the developmental and ecological relational aspects involved in the systemic organisation of reproductive processes (Etxeberria, 2023; Etxeberria & Cortés-García, 2026). From the organismal perspective, menstruation is understood as embedded in broader developmental processes and intra- and inter-organismic interactions, rather than an isolated function (Strassman 1996; Emera et al., 2012; Critchley et al., 2020). Three main factors explored in the context of menstruation evolution: the coordination of ovarian and uterine cycles, the emergence of the novelty of spontaneous decidualization, and ecological relational influences, help illustrate this organismal view. Together, they construct a comprehensive framework that reinterprets menstruation, and articulate a new narrative emphasizing its organizational and relational organismal significance.

Within this framework, the evolution of spontaneous decidualization is understood to have occurred only in lineages that had already evolved spontaneous ovulation. The latter provided the hormonal and regulatory conditions necessary for the former to arise; in other words, spontaneous decidualization did not emerge independently of the internal cyclicity established by spontaneous ovulation. Recognizing this sequence helps clarify how key reproductive innovations became integrated within the organismal system.

### The evolution of two cycles: ovarian and uterine

The key evolutionary changes associated with menstruation involve the ovarian and uterine cycles, which together constitute the menstrual cycle. This cyclicity must be understood from an organismal perspective, one that views physiological rhythms as emerging from the integrated functioning of the whole organism, rather than as isolated or mechanistic repetitions. In this view, physiological processes such as ovulation or endometrial renewal are not independent events but coordinated components of a systemic reproductive organization. The organismal approach therefore provides the conceptual link between evolutionary explanations and the physiological mechanisms through which menstruation takes place. To grasp the significance of these developments, it is essential to examine how both ovarian and uterine cyclicity have evolved.

A notable shift in this evolutionary trajectory is the transition from induced to spontaneous ovulation. Most mammals regulate reproduction through estrous cycles triggered by environmental factors, such as seasonal cues like the length of daylight or temperature, or by interactions with partners, including exposure to male pheromones or copulation (Pavliçev & Wagner, 2016). By contrast, menstruating species undergo spontaneous ovulation: a more autonomous, hormonally driven, and cyclic pattern of ovulation independent of immediate external stimuli. Physiological changes during both the estrous and menstrual cycles are often comparable, given their shared response to ovarian steroid hormones, the key difference lies in their regulation. The estrous cycle is externally regulated, whereas the menstrual cycle follows mostly an inherent cyclical rhythm. This shift represents a key evolutionary change, introducing year-round breeding and greater flexibility in reproductive timing.

Organisms that have shifted from estrous-based reproduction to a menstrual pattern typically exhibit concealed ovulation, allowing for year-round breeding. Rodents offer a valuable comparative framework, given the diversity in their reproductive strategies. In the common mouse, ovulation is induced by environmental factors. Following ovulation, progesterone levels drop and the endometrial lining is reabsorbed rather than expelled; thus, there is no uterine bleeding. In contrast, the spiny mouse, a rare menstruating rodent, exhibits spontaneous ovulation and uterine bleeding, making it a critical model for studying the evolution of menstruation (Bellofiore & Evans, 2019; Bellofiore et al., 2018a, 2018b).

Spontaneous ovulation established the hormonal and regulatory conditions under which spontaneous decidualization could evolve, and the two traits now co-occur in all menstruating species. However, their presence alone is not sufficient for menstruation to arise: some species display both spontaneous ovulation and decidualization yet do not menstruate. In these cases, the decidualized layer of the endometrium is reabsorbed rather than shed through bleeding. Menstruation thus evolved only in certain lineages where these processes became integrated with additional vascular and inflammatory dynamics that favored tissue breakdown and repair.

This transition marks a major reorganization of the uterine cycle itself. In species where decidualization depends on embryo implantation, the endometrial response is induced only after fertilization. In menstruating species, by contrast, decidualization occurs spontaneously as part of a regular cycle, independent of the presence of an embryo. This process involves the transformation of endometrial fibroblasts (connective tissue cells found in the supportive layer of the uterine lining or stroma) into decidual cells, a key step in preparing the uterine environment for potential implantation (Gellersen et al., 2007). Its evolution entailed significant physiological changes, including metabolic, immune, and vascular changes, that illustrate a tightly integrated relationship between reproductive function and broad organismal processes.

### Spontaneous decidualization as an evolutionary innovation

Recent advances in evolutionary developmental biology have emphasized that decidualization should be understood as an organismal evolutionary novelty, one that plays a central role in shaping both eutherian pregnancy and the emergence of menstruation (Chavan et al.,^2016^). Among eutherian mammals, reproductive traits vary widely. In groups with spontaneous decidualization, such as catarrhine primates, this trait is often associated with highly invasive placentation (a form of placental development in which fetal tissues penetrate deeply into the uterine lining) alongside increased uterine vascularization, and distinct hormonal mechanisms that initiate labor (Mika et al., 2021, p. 2). Spontaneous decidualization involves a notable influx of immune cells and vascular remodelling that help stabilize the endometrium and regulate tissue shedding during menstruation. By contrast, primates with non-invasive placentation typically do not exhibit spontaneous decidualization.

This evolutionary innovation is thought to have emerged as an adaptive response to the demands of invasive placentation (Jarrell, 2018). Decidual cells are thought to have evolved to mediate intra-actions^5^ between maternal physiology and the developing embryo, play a central role in this process (Wagner et al., 2019). In groups like the mouse, which do not menstruate naturally, decidualization can be experimentally induced, but tissue breakdown only occurs after progesterone withdrawal (Evans & Salamonsen, 2012). This suggests that menstruation emerged from evolutionary changes in endometrial cells that prevent reabsorption of the tissue in the absence of implantation. This transition brought about a cascade of physiological changes in menstruating organisms, influencing key physiological features now closely tied to the menstrual cycle. These include metabolic, immune, and vascular changes, highlighting the complex, system-wide interactions shaped by the evolutionary history of menstruation.

Taken together, these insights indicate that menstruation is not best understood as an independently selected trait, but rather as a consequence of spontaneous decidualization (Critchley et al., 2020; Emera et al., 2012; Ng et al., 2020), an evolutionary innovation rooted in the physiological and developmental demands of maternal–fetal intra-actions during pregnancy. The endometrium has been described as “a unique, dynamic tissue that is cyclically shed, repaired, regenerated and remodelled, in preparation for embryo implantation” (Evans et al., 2016). Menstruation, within this framework, serves as a reparative process when implantation does not occur. The evolution of spontaneous decidualization has been attributed to two complementary explanations. First, it may have arisen as a protective or collaborative interface for the gestating organism, especially in species with highly invasive placentation. Second, it may have a role in embryo selection, as decidualized stromal cells are more capable of detecting and eliminating compromised embryos (Alvergne & Högqvist Tabor, 2018). These explanations highlight the importance of developmental and structural processes in evolutionary biology, while also emphasizing the active agency of gestators in reproduction, rather than portraying them as mere passive containers. This reflects the fact that decidualization involves active physiological regulation by gestators, whose tissues detect, select and respond to embryonic signals, shaping the implantation and pregnancy outcomes (Nuño de la Rosa et al., 2021).

Moreover, parallels between menstruation and parturition further inform our understanding, as both processes involve inflammatory responses and are regulated by progesterone (Pavličev & Norwitz, 2018). This study emphasizes that key features of human pregnancy—such as decidualization, uterine inflammation, contractions, and endometrial shedding—are present in the menstrual cycle, even in the absence of pregnancy. Such insights encourage further research into decidualization outside the context of gestation, potentially shedding light on pregnancy-related complications. Notably, the shared inflammatory nature of menstruation and childbirth was recognized as early as the nineteenth century (Finn, 1986). Exploring these morphological and physiological parallels can enhance our understanding of both menstrual function and reproductive health.

### Ecological and anatomical factors in menstruation

A central feature of the organismal perspective is its attention to the agential exchanges between organisms and their environments. This view foregrounds how physiological processes like menstruation evolve from integrated, reciprocal relationships between internal and external conditions (Etxeberria & Cortés-García, 2026). From this standpoint, ecological and energetic contexts are not merely background constraints but active components in shaping reproductive physiology.

Ecological factors, including nutrition and resource availability, significantly influence the evolution of menstruation. Diet appears to play a central role in explaining why spontaneous decidualization evolved in some species but not in others (Bellofiore et al., 2018a, 2018b, 2019). Species capable of sustaining the energetic cost of maintaining a hormonally primed endometrium year-round would have been more likely to evolve spontaneous decidualization, whereas in energetically constrained or strongly seasonal environments, induced cycles remained adaptive. In species with estrous cycles, births are often timed to coincide with periods of food abundance, ensuring sufficient energy availability to support the demands of lactation. In species exhibiting spontaneous decidualization, maintaining a diet sufficient to meet the metabolic demands of the decidual response becomes particularly crucial, especially in environments where food availability is unpredictable. This suggests that ecological stability and energy balance acted as permissive factors for the evolution of spontaneous decidualization and, by extension, menstruation.

This ecological perspective also intersects with anatomical variation, particularly in uterine morphology, potentially linked to the evolution of menstruation (Pavličev & Wagner, 2022). Morphological differences, such as simplex, bicornuate, and double-chambered uteri, are observed both across and within species, including in humans (Gruber & Schlaff, 2021). Although a correlation between simplex uterine morphology and menstruation has been proposed, it is challenged by findings such as the menstruating spiny mouse, which possesses a double uterus similar to other rodents. These patterns indicate that uterine form alone does not determine menstruation; rather, it interacts with ecological and metabolic conditions that shape endometrial dynamics.

The evolution of menstruation may have triggered a cascade of physiological changes in menstruating organisms, shaping not only reproductive aspects related to the menstrual cycle but also influencing interconnected metabolic, immune, endocrine, and vascular factors (Emera et al., 2012; Jarrell, 2018). In this framework, the organism is seen as a complex, integrated whole, inherently connected to its environment and other organisms, and uterine structure must be viewed in the broader ecological context that shapes reproductive adaptations.

Taken together, these findings underscore a major rethinking of menstruation, not as a discrete adaptation, but as a systemic feature rooted in reproductive complexity. Insights discussed in this section suggest that menstruation is not merely a straightforward functional change, but a complex, multifaceted outcome shaped by a combination of hormonal, immune, metabolic, and ecological factors.

While byproduct hypotheses interpret menstruation as a secondary consequence of another selected trait, such as spontaneous decidualization, the organismal perspective shifts the focus from isolated traits to systemic organization. It understands menstruation as part of a broader physiological network that emerges at specific developmental stages, rather than as a discrete function or evolutionary byproduct. This explanatory shift moves beyond the functional/byproduct dichotomy, emphasizing the integration of hormonal, immune, and metabolic processes within reproductive life histories. In doing so, it aligns evolutionary accounts with a more organism-centered and systemic view of adaptation.

By adopting an organismal perspective, we can more fully appreciate the intricate interdependence of these elements in the evolutionary history of menstruation. This broader view contributes more effectively to evolutionary medicine than the traditional functionalist approach associated with DM, as it is grounded in proximate biological properties that can be potentially targeted or modified through various health interventions. If the question is whether menstruation is functional, the organismal perspective cautions against isolating it from the broader network of physiological systems, implying that attempts to identify a single, discrete function may be misguided. However, if the question is whether menstruation is adaptive in the sense of not being a useless byproduct, this perspective supports the conclusion that it is indeed an integrated and meaningful aspect of the physiology of organisms that menstruate.

## Reframing menstrual pathologies through an evolutionary perspective

The evolutionary perspective definitively reframes menstruation as a complex physiological process that is adaptive at an organismal level. For too long, menstruation has been mischaracterized as an anomaly, viewed as a deviation from medical standards historically grounded in male bodies and androcentric conceptions of health. This entrenched bias has not only distorted scientific understanding but also obscured the biological significance of menstruation.

In this section, we build on the organismal perspective to examine how it deepens our understanding of menstruation in the context of health and disease. Central to this approach is the recognition that menstruation is a multifaceted evolutionary trait with implications for a wide range of physiological processes, including, but not limited to, the reproductive. To challenge persistent negative stereotypes, we take into account insights from social, feminist, gender-based, and physiological perspectives (Bobe l et al. 2020; Clancy, 2023), which together offer a more holistic and inclusive understanding of menstruation’s role, particularly in the context of medical research and clinical care.

Menstrual physiology is closely tied to the endocrine system, vascular regulation, and patterns of blood loss (Jarrell, 2018). While these aspects are relevant in understanding, diagnosing and treating menstrual disorders, the evolutionary perspective adds crucial context by addressing the origins and adaptive purposes of such traits. Rather than viewing heavy menstrual bleeding solely as a pathology, this perspective invites consideration of whether it may have once conferred adaptive advantages—such as enhancing uterine receptivity or clearing non-viable embryos—before becoming maladaptive in present contexts. In this way, an evolutionary perspective broadens our understanding of normality and disease by situating physiological variation within an evolutionary developmental framework, rather than treating it solely as deviation from a biomedical ideal.

The evolutionary foundations of menstruation also help clarify how menstrual cycles influence health. As Alvergne and Högqvist Tabor (2018) argue, evolutionary insights are essential not only for addressing current health challenges but also for anticipating how modern environments interact with ancestral adaptations. Likewise, Jarrell (2018) contends that an evolutionary framework can shift research and clinical focus from symptom-based treatment toward uncovering biological causes rooted in history. Importantly, menstruation’s cyclical nature, driven by hormonal and immune changes, impacts multiple aspects of health and disease beyond reproduction. As Jarrell notes, “awareness of the significance and evolution of menstruation provides an opportunity to explore the treatment and future research opportunities of a wide variety of experiences of the human condition” (2018, p. 23).

To examine the broader health implications of menstruation through an evolutionary lens, we focus on three interrelated dimensions where evolutionary medicine offers critical insight. First, the cyclical nature of immune modulation across the menstrual cycle plays a key role in shaping systemic health outcomes, with inflammatory patterns influencing susceptibility to disease and overall immune function. Second, the structure and function of the endometrium, deeply shaped by evolutionary and reproductive pressures, provides an important context for understanding both normative menstrual physiology and common pathological conditions (Etxeberria, 2016). Finally, modern environmental exposures, such as endocrine-disrupting chemicals, may generate mismatches between evolved biological systems and current ecological contexts, contributing to emerging health challenges. Together, these dimensions underscore how menstruation, when viewed from an organismal evolutionary perspective, is not a discrete reproductive event but a complex dynamic physiological process, embedded in the organism’s adaptive history and current ecological context. Rather than framing menstruation as inherently problematic, the evolutionary organismal perspective helps recognize it as a multifaceted evolutionary trait that deeply influences health beyond reproductive goals. It is a vital biological phenomenon shaped by complex intra-actions between internal physiology and the external environment. This framework opens the way for research, clinical care, and health policy that acknowledge biological diversity and better support the varied experiences of those who menstruate.

### The cyclicity of inflammatory patterns and immunity in menstruators

Recent scientific findings increasingly support the view that the menstrual cycle is a biologically and evolutionarily significant process, shaped in part by cyclical inflammatory and immune activity. These recurring immunological changes provide important insights into both the evolutionary role of menstruation and the health vulnerabilities it may entail. One influential hypothesis suggests that such inflammatory patterns evolved to allow gestators greater control over embryo selection, thereby increasing reproductive efficiency through the early elimination of non-viable embryos (Alvergne & Högqvist Tabor, 2018). In this context, menstruation may be understood as a byproduct of the evolution of the uterine cycle; it is considered to have arisen in response to alleged fetal-gestator genetic conflict over resource investment, particularly intense in humans due to the invasiveness of the trophoblast and the high rate of embryonic genetic abnormalities (Elliot & Crespi, 2006; Emera et al., 2012).

One key hypothesis holds that the evolution of spontaneous decidualization, closely linked to the emergence of cyclic menstruation, represents a major evolutionary adaptation, one that optimises the trade-off between maternal selectivity and embryonic receptivity (Alvergne & Högqvist Tabor, 2018; Abrams & Miller, 2011; Mitchell et al., 2022). These evolutionary adaptations, while beneficial in ancestral environments, may introducee health vulnerabilities in modern contexts, where increased cycle frequency and novel lifestyle factors can lead to inflammatory dysregulation and related pathologies.

Beyond reproductive outcomes, cyclical immunity has broader implications for general health. Alvergne & Högqvist Tabor (2018) stress the menstrual cycle’s implications for general health beyond reproduction, as cyclical immunity also affects non-reproductive health. The immune function varies cyclically in response to hormonal changes across the menstrual cycle. Each phase of the cycle is characterised by distinct hormonal profiles, which in turn modulate immune responses.

For example, the activity of immune cells such as natural killer (NK) cells varies across cycle phases, modulating the body’s response to pathogens and environmental stressors. Attention has turned to the role of cyclical immunity, its role in the development of autoimmune diseases and the potential mismatch between evolved immune patterns and current environments (Rook, 2013). Epidemiological patterns show a higher incidence of autoimmune conditions during the reproductive years, particularly in association with physiological processes related to menstruation (Desai & Brinton, 2019). One line of inquiry attributes this to hormonally mediated “windows of immune tolerance” during the cycle. During these phases, immune vigilance is temporarily reduced to protect potential pregnancies, but this downregulation may also increase susceptibility to dysregulated immune responses. In such windows, the immune system may tolerate or fail to regulate targets it would otherwise eliminate, potentially triggering or exacerbating autoimmune activity. These dynamics raise important questions about the compatibility of evolved immune patterns with contemporary life. Keestra et al. (2021) argue that a mismatch may now exist between the ancestral ecological settings in which immune adjustments evolved and modern patterns of extended hormonal exposure, driven by changes in reproductive timing, contraceptive use, and environmental stressors. These cyclical immune adjustments evolved to balance maternal tolerance and foetal protection from immune rejection and may become maladaptive, contributing to chronic immune disorders among menstruating individuals**.** In addition, the idea that immune cyclicity is evolutionarily attuned to environmental and social conditions invites broader philosophical reflection. Some scholars suggest that menstrual patterns may be understood not merely as internal physiological rhythms, but as processes through which the organism calibrates itself to external realities. This idea aligns with organismal and systems-based perspectives in philosophy of biology, which view bodily processes as dynamic interfaces between internal regulatory mechanisms and broader ecological or cultural environments. A particularly illustrative case comes from sports science. Until the 1980s, training protocols were largely modelled on male physiology, assuming uniform responses and ignoring the effects of menstrual cycles. However, recent research underscores the importance of accounting for menstrual cycle variations when designing training regimens for menstruating athletes (McNulty et al., 2020). Acknowledging menstrual cyclicity not only improves performance outcomes but also challenges long standing assumptions about physiological “norms” (Alvergne & Högqvist Tabor, 2018), and reinforces the value of inclusive, organismal research approaches.

### The needs and complexities of the endometrium repair and renewal

Many contemporary menstrual disorders and pathologies can be traced to the physiological demands placed on the uterus, particularly the endometrium, and to the evolutionary processes that shaped its current form and function (Maybin & Critchley, 2015). Studying the evolutionary and developmental history of cells, tissues, and organs provides not only historical context but also a valuable explanatory framework for understanding patterns of health and disease in menstruating individuals (Mika et al., 2021).

One notable feature in this context is the comparatively large volume of menstrual bleeding observed in humans (Jain et al., 2022; Rogers-LaVanne & Clancy, 2021). This characteristic, not uniform across species, has been linked to the evolutionary enlargement of the human uterus, as the relatively large size and shape of the uterus support a more substantial build-up of endometrial tissue during the menstrual cycle. In contrast to species where the endometrial lining is reabsorbed or only lightly expelled, humans undergo a more pronounced shedding process. The volume of blood loss appears proportional to both the uterine dimensions and the thickness of the endometrium (Catalini & Fedder, 2020; Emera et al., 2012). Although the evolutionary rationale remains debated, one prominent hypothesis suggests that heavy menstruation promotes the efficient clearance of excess endometrial tissue, thereby preserving uterine receptivity for future embryo implantation.

These evolutionary and anatomical considerations become especially pertinent in the context of reproductive challenges and assisted reproductive technologies. Recurrent pregnancy loss, typically defined as two or more miscarriages before the 20th week of gestation, has been associated with impaired endometrial stem cell function. Such impairments may result in inadequate endometrial thickness or reduced receptivity, thereby limiting successful embryo implantation. Further research has identified accelerated stromal senescence, stem cell deficiencies, and defective decidualization as contributing factors, all of which diminish the endometrium’s capacity to differentiate and support pregnancy (Evans et al., 2016).

Furthermore, endometriosis exemplifies the complex interplay between evolved endometrial features and pathological outcomes (Ruiz-Alonso et al., 2012). This condition, characterised by the growth of endometrial-like tissue outside the uterine cavity, involves multifactorial causation, encompassing genetic, hormonal, and immunological components. A prevailing theory suggests that some individuals possess highly proliferative or invasive endometrial stem cells, which may increase the likelihood of implantation and growth of endometrial-like tissue in areas outside the uterus, such as the peritoneum. Once established, this ectopic tissue continues to respond to hormonal cues, triggering inflammation, scarring, and painful lesions (Laganà et al., 2019). This process can result in a range of symptoms, including chronic pelvic pain, painful menstruation, pain during sexual intercourse, and in some cases, fertility problems.

Given these properties of the endometrium, some researchers advocate for the therapeutic suppression of menstruation to manage conditions like severe dysmenorrhea or to prevent chronic pelvic pain (Jarrell, 2018). However, while this approach can be effective in symptom reduction, this strategy does not address the underlying evolutionary and physiological context. In response, a growing number of scholars now argue that lifestyle changes may offer a more sustainable approach to preventing or alleviating these conditions (Allen, 2025). These possibilities are discussed in the next subsection.

### Lifestyle mismatches and environmental threads

As introduced earlier, a growing body of research literature emphasizes the importance of recognising that menstruation evolved under environmental and social conditions that no longer prevail. To fully understand how the organisational characteristics of menstruating organisms may give rise to specific pathologies today, it is essential to consider how modern circumstances, particularly in industrialised societies, differ from ancestral environments. Although menstruation is a long-standing and deeply embedded aspect of human biology, it was shaped in relation to distinct ecological and lifestyle pressures. The menstrual cycle, while deeply embedded in the biological organization of the human body, does not operate in isolation. It remains responsive to both internal (endogenous) and external (exogenous) cues, highlighting the dynamic relationship between organism and environment. This responsiveness is particularly evident when considering how contemporary environmental and lifestyle shifts impact menstrual health.

In Sect. 4, we discuss Beverly Strassman’s contribution to the function-versus-byproduct debate on menstruation. Her later work illustrates the evolutionary mismatch perspective by emphasizing the contrast between the evolutionary context in which menstruation arose and the radically different conditions of contemporary life. In her long-term field study of a pre-industrial society, Strassman found that individuals typically experienced around 128 menstrual cycles over the course of their lifetimes. By contrast, menstruating individuals in industrialised societies now experience approximately 450 cycles. This dramatic increase is largely attributable to changes in reproductive patterns, including later menarche, lower parity, fewer and shorter periods of breastfeeding, and later age at first pregnancies. The key insight is that greater exposure to endogenous oestrogen, resulting from increased cycle frequency, may be linked to a higher incidence of hormone-related diseases, such as breast cancer (Strassman, 1997). Strassman’s work thus underlines how evolutionary mismatches, particularly cumulative hormonal exposure and its associated risks, have menstrual health implications. Strassman’s perspective stresses how modern lifestyles introduce a number of variables such as changes in diet, levels of physical activity, and exposure to synthetic chemicals, that that may further intensify this mismatch. These influences reinforce the view that contemporary menstrual patterns are not determined by biology alone, but are also shaped by social, environmental, and technological transformations that interact with, and sometimes disrupt, evolved physiological systems (Goldstuck, 2020; Keestra et al., 2021; Maric-Bilkan et al., 2014; Vercellini et al., 2024).

Among the most concerning of these disruptions are endocrine-disrupting chemicals (EDCs), which can interfere with menstrual regularity and overall reproductive health (Clancy, 2023). These substances, often synthetic chemicals, can mimic, block or alter the natural activity of hormones such as oestrogen and progesterone. Since these hormones are central to ovulation, endometrial development, and menstruation, exposures to EDCs can result in menstrual irregularities, which may result in difficulties conceiving, pregnancy complications, or an increased risk of certain health conditions (Clancy, 2023). In light of these concerns, increasing attention has been paid to modifiable lifestyle factors that may mitigate some of these environmental threats. Public health recommendations concerning individuals during the menstruating stage often include not only general health measures, such as balanced nutrition, adequate sleep, and physical activity, but also specific avoidance of products containing harmful substances, including certain plastics, creams, and cosmetics.

While such recommendations benefit the general population, they are particularly urgent for menstruating individuals, whose cyclical physiology may make them more sensitive to environmental inputs (Jasienska, 2013). Recognising this physiological sensitivity allows for a more informed approach to health, attentive to individual biological rhythms and environmental exposures.

Rather than viewing menstruation solely through the lens of dysfunction or disease, it should be understood as part of a complex biological and evolutionary phenomenon, with consequences for reproductive function, biological organisation, and health. This section has outlined three interrelated domains in which evolutionary perspectives on menstruation can enrich our understanding. Cyclical immune patterns shape physiological organisation, particularly immune function; the dynamic processes of endometrial repair and renewal are essential for understanding menstrual disorders; and lifestyle and environmental exposures, including nutrition and chemical pollutants, must be considered to support physiological balance.

The central argument of this paper has been that an evolutionary perspective can contribute to overcoming the stigma attached to menstruation by framing it as a biologically significant and ecologically situated phenomenon. While functionalist accounts of its evolution have contributed important insights, they often overlook the developmental and environmental complexity of menstrual biology. We have proposed that an organismal approach, one that emphasizes the relational dynamics among physiology, development, and environment, not only deepens our understanding of menstruation’s evolutionary history but also offers a more effective framework for identifying the sources of menstrual pathologies. For these reasons, evolutionary medicine should adopt an organismal-relational perspective as central to its study of menstruation.

## Conclusions

Questions about the significance of menstruation, such as why it exists or whether it is necessary, call for an evolutionary perspective. In this paper, we examined major responses to these questions, which, during the 1990s, focused on the debate between functionalist and by-product accounts. While functionalist arguments have provided heuristics, their relevance to medicine remains limited. Over time, evolutionary thinking expanded to incorporate organismal approaches that draw from evolutionary developmental biology and environmental interactions. These perspectives consider developmental mechanisms, structural and comparative aspects of the reproductive system, and environmental influences, offering a richer understanding of how menstrual physiology has evolved across species. This shift compels us to view menstruation not as an isolated trait, but as a part of an evolving and integrated reproductive system.

Although feminist scholarship has long critiqued the stigma attached to menstruation, even prominent voices have struggled to fully embrace it in positive terms. We argue that menstruation should not be regarded just as an unavoidable physiological fact but as a meaningful evolutionary innovation integrated into a complex system. Its cyclical nature plays a crucial role in immune regulation, endometrial renewal, and reproductive agency, with implications extending beyond reproduction alone. Rather than framing menstruation as a flaw or pathology, it should be understood as a fundamental aspect of the biological constitution of certain species, including humans. Viewing menstruation in this way enables critical engagement with health-related issues and encourages a more inclusive, scientifically grounded account of biological normativity.

From the perspective of philosophy and the recent history of science, menstruation can be reframed by addressing how it evolved and what interactive roles it plays, challenging societal values that lead to stigma and pathologization. At the same time, it is essential to acknowledge that menstruation can be painful and difficult for many, and that the medical field has often failed to adequately address these experiences. The implicit suggestion that menstrual discomfort should be passively accepted as a natural burden must be critically examined. Our aim here has been to highlight the systemic and complex dynamics of the processes involved in the evolution of menstruation, and to clarify how these features may give rise to medical conditions that require appropriate attention and care.

The complexity of menstruation calls for sustained theoretical and philosophical engagement. From this standpoint, we have proposed that evolutionary frameworks in medicine can help identify at least three broad classes of menstrual disorders: those linked to immune and hormonal cyclicity; those involving the regenerative properties of the endometrium; and those emerging from mismatches between contemporary lifestyles and ancestral conditions. Recognizing these dynamics deepens our understanding of menstrual pathologies and highlights regulatory participation of menstruators in reproductive outcomes, as exemplified by the endometrium’s active role in embryo selection and receptivity. Finally, an organismal-relational approach to menstruation underscores its role in regulating health across multiple domains, highlighting the need to attend not only to its physiological organization, but also to the broader ecological and social conditions in which it unfolds.

## Supplementary Information

Below is the link to the electronic supplementary material.

Supplementary Material 1

## Footnotes

1 This usage aligns with current trends in biomedical and feminist literature that seek to describe reproductive traits without assuming categorical equivalence between sex and physiological function. Our approach follows recent discussions in philosophy of science on the contextual use of sex reference classes in clinical and experimental research, and the arguments of authors such as Sarah Richardson (2022) and Aja Watkins & Marina DiMarco (2025) for moving beyond binary classifications. In the case of menstruation, we treat it as a reference class with ambiguous boundaries, unclear as to how far back or forward to include past, present, or potential menstruators, and not coextensive to the conventional categories of woman or female. However, where necessary, we retain the terms; also to reflect the terminology used in the literature we cite. We thank our reviewer and editor for suggesting that we clarify this aspect.

2 Decidualization is a transformation of uterine cells that prepares the uterus for potential pregnancy. Those cells constitute an evolutionary novelty; in some species, the process occurs cyclically even without pregnancy, playing a key role in menstruation (Chavan et al., 2016 ).

3 Standard definitions of menstruation commonly allude to the "regular renewal of the lining of the uterus" (Renfree 2012, 1), the "cyclical shedding of the endometrium triggered by falling progesterone levels" (Alvergne & Högqvist Tabor, 2018, p. 401), or the "phenomenon of uterine bleeding and shedding between successive ovulations" (Profet, 1993, p. 336). All three definitions refer to the shedding of the uterine lining, though they differ in their focus on factors like ovulatory timing and hormonal regulation.

4 While the terms “without menstruation” and “male” are not coextensive, the concept implies that the organism viewed as neutral and free from endocrine, immune or other complexities is typically perceived as male.

5 We use "intra-action" rather than "interaction," following Karen Barad’s terminology (Barad, 2007), to emphasize that entities do not preexist their relations but emerge through relational entanglements.
