# Supplementary material for: Reframing the significance of menstruation: evolutionary insights from an organismal-relational perspective
Source: Hist Philos Life Sci. 2025 Dec 16;48(1):2. doi: 10.1007/s40656-025-00710-5 (PMC12708708; doi:10.1007/s40656-025-00710-5)
Supplement: Supplementary file 1 — Supplementary Material 1 [file 40656_2025_710_MOESM1_ESM.docx]

Dear editor, Prof. Adrian Currie:

Thank you very much for your message and for accepting our manuscript *“Reframing the significance of menstruation: evolutionary insights from an organismal-relational perspective”* for publication pending minor corrections. We appreciate the opportunity to revise the paper in line with the reviewers’ final suggestions, and we have carried out all the requested modifications according to their advice. We also confirm that we have followed the submission instructions provided and uploaded the editable source files as required.

We are very pleased that our paper has been accepted for publication, and we would like to thank you and the reviewers for your careful and constructive work. The detailed comments from both reviewers, including also those raised by Reviewer 2 in the second revision, have helped us to substantially improve the clarity and coherence of the text.

We have carefully addressed all the remaining questions and provided detailed responses below, following the structure of the letter and report we received. Each response indicates how the corresponding point has been addressed in the revised manuscript. We also attach two versions of the revised manuscript: one with tracked changes and another with all changes accepted.

We will, of course, wait to see if any further revisions are required. Once the manuscript is ready for final processing, we would like to make a last revision to include the references that were omitted for anonymity, as well as the acknowledgements section.

In addition, we would like to publish this article as *open access* under the agreements established by our institution, the University of the Basque Country (EHU).

Kind regards,

Arantza and Ainhoa
